# Coupling of Microalgae Cultivation with Anaerobic Digestion of Poultry Wastes: Toward Sustainable Value Added Bioproducts

**DOI:** 10.3390/bioengineering8050057

**Published:** 2021-05-04

**Authors:** Rajinikanth Rajagopal, Seyyed Ebrahim Mousavi, Bernard Goyette, Suman Adhikary

**Affiliations:** Sherbrooke Research and Development Center, Agriculture and Agri-Food Canada, 2000 College Street, Sherbrooke, QC J1M 0C8, Canada; ebrahim.mousavi65@gmail.com (S.E.M.); bernard.goyette@canada.ca (B.G.); suman.adhikary.buet@gmail.com (S.A.)

**Keywords:** anaerobic digestion, ammonia, bacteria consortia, bioproducts, *Chlorella vulgaris*, microalgae

## Abstract

Third generation biofuels and high-value bioproducts produced from microalgal biomass have been considered promising long-term sustainable alternatives for energy and/or food production, potentially decreasing greenhouse gas emissions. Microalgae as a source of biofuels have been widely studied for bioethanol/biodiesel/biogas production. However, critical research is needed in order to increase the efficiency of microalgae production from high-N agri-waste, not only for biofuels but also for bio-based products, and thus enhance its commercial viability. The growth in the poultry industry has led to increased chicken manure (CM), which are rich in ammonia, phosphate, potassium, and other trace elements. These constituents could be used as nutrients for growing microalgae. In this research, a two-stage (liquid–solid) anaerobic digester treating CM at 20 ± 1 °C was performed, and liquid digestate (leachate) obtained after the digestion process was used as a substrate to grow the microalgal strain *Chlorella vulgaris* CPCC 90. Considering the high-N content (NH_3_-N: 5314 mg/L; TKN: 6197 mg/L) in liquid digestate, different dilutions were made, using distilled water to obtain viz. 10%, 30%, 50%, 70%, 90%, and 100% of the digestate concentrations for the microalgae cultivation. Preliminary results showed that *Chlorella vulgaris* CPCC 90 was able to grow and utilize nutrients from a 10% diluted CM digestate. Future research is underway to enhance microalgal growth at higher digestate concentrations and to optimize the use of microalgae/microalgae-bacteria consortia for better adaptation to high-N content wastes. An AD-microalgae coupling scenario has been proposed for the circulation bioeconomy framework.

## 1. Introduction

Canada’s commercial chicken and turkey meat production was over 1.43 billion kilograms in 2018 and the demand for poultry meat production as the most consumed meat animal protein source is growing at an average rate of around 2.3% annually [1]. In order to process this high volume of manure, appropriate manure management strategies are necessary. The potential impact of disposing untreated chicken manure (CM) in the environment is one of Canada agriculture’s major challenges, considering its high volume and its concentrations. The treatment of poultry wastes has gained attention for its environmental impact mainly due to its high organic and high nutrients, including nitrogen (N) and phosphorus (P) loads. Specifically, the excess content of nitrogen in CM contributes largely to environmental pollution through nitrate discharge to surface and ground water bodies, and ammonia or NOx emissions to the atmosphere [2].

A broad range of CM treatment technologies have been reported, such as anaerobic digestion, direct combustion, extruding, and rendering [3,4,5,6]. Among these technologies, anaerobic digestion (AD) can play an important role in the management of CM, as, unlike other techniques, it is comparatively a low-cost process for livestock farming and relatively easy to apply in farms [3]. AD process can provide an alternative to land application through solving specific problems, such as odors, pathogens, water pollution, greenhouse gas (GHG) emissions, and phosphorus and heavy metal contamination of soils, to some magnitude. This method has rarely been industrialized for raw CM treatment due to the problems associated with its high amounts of total solids (TS) and ammonia concentrations, which can inhibit methanogenic archaea [7]. Despite the effectiveness of the AD process, land application of CM digested materials still contain organics and excess nutrients that could lead to ammonia emissions, groundwater pollution or eutrophication of lakes as a result of rainwater runoff. Recovering nutrients (N, P) from nutrient-rich CM digestate after the AD process could solve the aforesaid problem.

Production of photosynthetic micro-organisms, or microalgae, has been the subject of interest in many recent studies due to its capability to grow and multiply rapidly by taking up various forms of nutrients from water, including N, P, and K, and yielding organic exploitable biomass [8,9,10]. Despite the promising advantages that microalgae proposes, in reality, its overall worldwide production is around 15,000 t/y annually [11], in contradiction with its potential demand. One major reason behind this low production is the necessity for large amounts of nutrients, including N and P, for mass production of microalgae. Approximately 5 t of N and 1 t of P are required to produce 100 t of microalgae [12]. This high amount of required nutrients limits microalgae mass production. Therefore, integrating microalgae production with other waste treatment technologies, such as AD, is becoming fascinating and vital for livestock sectors since it recovers valuable nutrients to be employed as new means of agricultural income. This integration offers additional benefits, such as the ability of microalgae to remove heavy metals, organic matter and inorganic nutrients [13,14], which provides cleaner effluent for further treatment stages leading to lowering the cost of wastewater treatment. It also provides a promising cost-effective option not only for tackling the high ammonia content issue but also providing long-term sustainable alternatives for energy/food production.

In addition to the environmental importance of microalgae cultivation, there are many studies and industrial reports describing the economic benefits of this practice through providing numerous applications, such as biofuels and biodiesel production, human food supplements, animal and aquaculture feed additives, bio-control of pathogens and pests, and soil treatment and fertilizer [15,16,17]. Among all these benefits, producing algal biofuel has gained the most attention by researchers and business investors. Producing biofuels from microalgae, however, still is not economically viable due to various hurdles, such as high costs associated with cultivation and harvesting per unit area, huge amounts of nutrients and supplements needed, efficient oil-extraction processes as well as physical–chemical conditions, which need to be carefully adjusted and monitored in order to increase the oil content of microalgae [16]. For this reason, an alternative approach must be considered for utilizing microalgae in farms in order to provide the basis for a circular bioeconomy. The utilization of AD effluent of animal waste as the sole nutrient source for producing microalgae has previously been reported. There is considerable literature on recovering anaerobically digested swine manure using microalgae [18,19,20,21,22]. A number of studies have reported the growth of microalgae from digested dairy wastewater [9,23,24]. Although there is extensive research on the utilization of dairy and swine digestate to grow microalgae, very limited research has been done on using CM digestate as a sole nutrient for microalgae growth [25,26]. Though several microalgal strains have been used to treat the diluted digestate, *Chlorella* sp. make the best candidate as they are widely cultured to produce food and biofuels, and are capable of both autotrophic and heterotrophic growth whenever an appropriate carbon source is provided [27]. Furthermore, the *Chlorella* sp. have a higher growth rate in the anaerobic-digested effluent (with 80–100 mg L^–1^ ammonia nitrogen) [9,28] and it also outperforms other species with regards to nitrogen removal [25]. It is evident that this approach has been studied for different forms of agricultural and animal wastes, while it has been rarely investigated for poultry waste. Poultry manure contains significant amounts of inorganic and organic P. At the same time, high concentrations of ammonia in CM digestate will disrupt the growth conditions for microalgal strains. In addition, the highly variable chemical composition of CM digestate makes it a more challenging medium to grow microalgae.

Thus, the coupling of microalgae cultivation with the anaerobic digestion of CM was proposed in this study for energy recovery and nutrient supply. AD would potentially function as a detoxification step prior to microalgae cultivation [29]. Organic pollutants, which are known to be toxic to algal growth, can be degraded during digestion; thus, a lower dilution ratio of the CM digestate (leachate) can be used for microalgae cultivation. Organic carbon in the CM digestate can also be recovered as methane-rich biogas, whereas the N and P in the digestate can compensate for the cost for algae cultivation. In this perception, the main objective of this work is to determine the feasibility of growing microalgae using a CM digestate (leachate) rich in ammonia, obtained from a laboratory scale, two stage (liquid–solid) anaerobic digester. Preliminary investigations have been conducted at the laboratory scale primarily to evaluate the adaptability of a microalgal strain to anaerobically digested CM, and to evaluate the tolerance of microalgae to the CM digestate. Furthermore, the proposed work, by coupling AD–microalgae cultivation, is expected to provide unique, science-based knowledge that can be used as an ideal agricultural waste bio-refinery model toward a circular bioeconomy concept to achieve substantial economic and environmental benefits, and therefore, reduce environmental risks associated with excess nutrients and GHG emissions.

## 2. Materials and Methods

### 2.1. Anaerobic Digestion: Feedstock, Inoculum and Experimental Set-Up

In this study, raw CM and, subsequently, the anaerobically digested liquid leachate were used as feed for AD and the microalgae cultivation process, respectively. The liquid portion of the digestate (leachate) was obtained from ongoing two-stage high-solid anaerobic-digestion (HS–AD) systems treating CM rich in solids and ammonia. For the HS–AD process, the raw CM was sourced from a small-sized poultry farm located in Farnham (Quebec province). The CM collected from a pile of waste litter, consisted of wood shavings as bedding. The liquid inoculum used in the start-up phase of HS–AD was obtained from our ongoing laboratory-scale liquid sequencing batch AD, adapted to CM leachate with high ammonia concentrations (5–7 TAN/L).

The experimental set-up consisted of two-stage (liquid–solid) anaerobic digesters (i.e., liquid inoculum reservoir coupled with the HS–AD system) for processing CM at 20 ± 1 °C. A set of digesters in triplicate with a total volumetric capacity of 40 L were operated in parallel. A set consisted of 2 digesters—one for the liquid inoculum reservoir and the other for HS–AD. The operational details of the two-stage AD process have been presented in our previous work [30]. The operational feasibility of the HS–AD process treating CM were experimented in 4 consecutive batch operations (cycles). For each operational phase, a cycle length of 70 d was maintained, and the organic load rate (OLR) was increased in a step-wise manner from Phase 1 to 4. At the end of each cycle of operation, the adapted inoculum was retained within the digesters for subsequent operation to expedite the digestion process. Characteristics of CM raw samples and liquid digestate (leachate) obtained after the HS–AD process from a typical batch operation are shown in Table 1.

### 2.2. Microalgae Cultivation Protocol

Due to the existence of high nitrogen concentration and pathogens in CM, it was critical to find a strain that has high adaptation capabilities to different environments. *Chlorella vulgaris* CPCC 90 was selected, as it has been reported to be a high adaptive strain and, also, it has high photosynthetic ability, which makes it an ideal candidate [15,22]. The microalgae *Chlorella vulgaris* was obtained from the Canadian Phycological Culture Centre, Waterloo, Canada. The strain was provided growth on Bold’s basal medium (BBM) (15 mL) and was stored at 4 °C.

In the next step, *Chlorella vulgaris* was transferred to a sterile Bold’s basal medium (BBM) supplemented with a vitamin solution to allow the propagation of the microalgal strain for further experiments. Sterile BBM was also obtained from the Canadian Phycological Culture Centre, Waterloo, Canada. To prepare the medium for each experiment, 10 mL of the concentrate medium plus 1 mL of Stock 5 solution was added to 989 mL of Mili-Q water. Then, the pH was adjusted to 6.8 with the addition of 1N HCl or 1N NaOH, if needed. Then, the medium was autoclaved and stored for further experiments.

Different dilutions of the liquid digestate were prepared for experimentation to investigate the impact of high N concentration. In this regard, the digestate was diluted using distilled water to prepare six different digestate concentrations viz. 10%, 30%, 50%, 70%, 90% and 100% (i.e., no dilution). Then, each sample was autoclaved at 121 °C for 20 min.

Growth conditions for all the experiments were adjusted as follows: temperature = 20 ± 0.5 °C, pH of the growth medium = 6.8, photoperiod = continuous lighting (cool-white fluorescent bulbs), agitation = 70 rpm, aeration = not needed (for small volume cultures as in our case), and inoculation ratio from original culture to sub-cultures = 1:10 ratio. Under these circumstances, 5 to 7 days were required for *Chlorella vulgaris* CPCC 90 to completely grow on sterile BBM media. However, this period could change according to the type of media and the growth conditions (for example, a higher temperature of up to 25 °C may lead to higher growth rates [31]).

**Figure 1:**
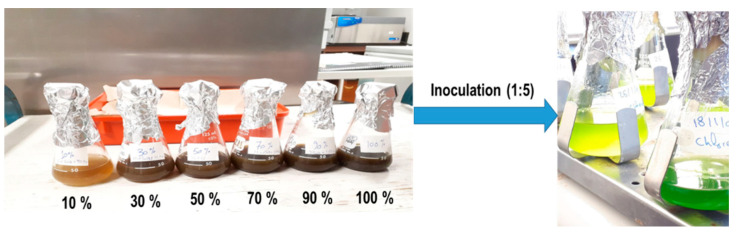
Various diluted samples of anaerobically digested CM (10 to 100%) used for microalgae cultivation experiments.

After obtaining enough growth of *Chlorella vulgaris* CPCC 90, the propagated strains were used to grow on the liquid digestate from the anaerobic biodigesters at a digestate–strain ratio of 1:5, as shown in Figure 1.

### 2.3. Analytical Methods

#### Physicochemical Parameters of CM Leachate

The raw CM and liquid digestate samples were analyzed for pH, alkalinity, total solids (TS), volatile solids (VS), total COD (TCOD) and soluble COD (SCOD) as per the standard methods [32]. The pH and alkalinity were measured using pH Mettler Toledo AG 8603, SevenMulti (Schwerzenbach, Switzerland) and Hach Lagne Sarl, Titralab AT1000 Series (Hach, Switzerland), respectively. COD was measured by using a closed reflux colorimetric method [32]. TKN and NH3-N were analyzed using a 2460 Kjeltec Auto-Sampler System (FOSS, Sweden) following the macro-Kjeldahl method [32]. Volatile fatty acids (VFAs) were determined using a Perkin Elmer gas chromatograph, model Clarus 580 (Perkin Elmer, Shelton, CT, USA), mounted with a DB-FFAP high-resolution column, but before the evaluation of VFAs, samples were conditioned according to the procedures mentioned by [33]. Samples collected from digesters were first centrifuged at 41× *g* for 15 min and filtered through a 0.22 µm membrane before being injected, and the injection volume was 0.1 µL. The biogas production and its composition were monitored regularly for both the liquid and solid digesters. The production rate was monitored every day using the wet tip gas meters, and its concentrations were analyzed thrice a week using a gas chromatograph (Micro GC 490, Agilent Technologies, Santa Clara, CA, USA) equipped with a thermal conductivity detector (TCD) and helium gas as the carrier gas at a flow rate of 20 mL/min. The injector and oven temperatures were 110 °C and 180 °C, respectively.

## 3. Results and Discussion

### 3.1. Performance of Two Stage High Solids Anaerobic Digestion

As mentioned in the previous sections, microalgae cannot grow directly on the untreated CM, as it contains high concentrations of ammonia as well as highly variable chemical compositions. Therefore, the feed for the microalgae needs to be processed. In this study, the raw CM was treated using a two-stage (liquid inoculum reservoir coupled with HS–AD) AD process, which was adopted as a pre-treatment for microalgae cultivation. The digesters were operated for a total period of 280 days in four batch operations (cycles), and each batch operation was conducted for a 70 d cycle. Operational parameters, such as organic loading rate (OLR), cycle length/treatment period, operating temperatures, recirculation–percolation rate and frequency, and the mode of operation, were controlled, as they have a direct influence on the performance of the two-stage AD process. In addition to this, the effect of ammonia concentrations on the digester’s performance was also given priority. The fundamental reason behind considering all the above-mentioned parameters was to have a stable AD operation. Subsequently, the digestate can be viably used to cultivate microalgae with minimal inhibitions. Furthermore, successful operation of the AD process could eventually generate more suitable digestate for microalgae production. Therefore, the operational stability in terms of biogas quantity, CH_4_ concentration, specific methane yield (SMY), NH_3_-N concentration, VFA, TS, VS and COD (both TCOD and SCOD) was monitored.

**Figure 2:**
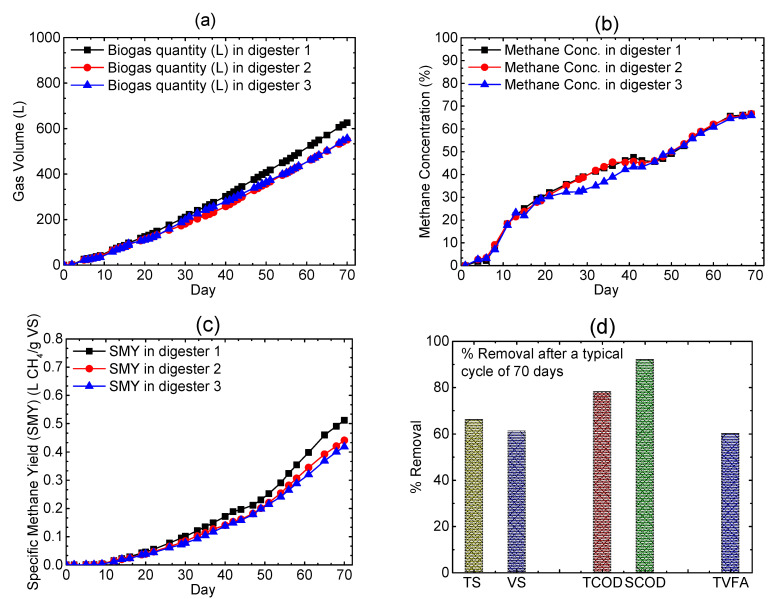
(**a**) Cumulative biogas quantity (L); (**b**) CH_4_ concentration profile; (**c**) specific methane yield (SMY); (**d**) percentage removal of TS, VS, TCOD and SCOD for a typical cycle length of 70 days.

An example of the results obtained from a typical cycle of operation is presented in Table 2, whereas Figure 2a–d depicts the performance of the digesters (in triplicate).

From Figure 2a,b, it is evident that the volumetric combined biogas production improved with time; their corresponding methane concentrations for all three identical digesters increased more or less steadily and eventually reached the plateau after 70 days. Thus, there is a clear indication of the smooth operation of the digesters, which has been further justified by the presence of very low amounts of free NH_3_-N i.e., 325 mg/L (data not shown here), which is within the threshold value of 500 mg/L [30]. Furthermore, SMY of 0.46 ± 0.05 indicates the richness in the methanogenic communities without any potential inhibitions. Another widely accepted indicator for digester stability is the total volatile fatty acids (TVFA) and Total Alkalinity (TA) ratio. Typically, the TVFA/TA ratio less than 0.8 indicates stable digester operation. In our study, data shows that TVFA/TA ratio remains well below 0.8, indicating that the digesters were operating favorably without the risk of acid accumulations. However, due to the fact that microbes are well acclimatized, there is no evidence of VFA accumulation (data not shown here) observed over that period, thus, they produced steady state methane production. It is clearly understood that the digesters were not facing any visible inhibition despite high ammonia and high solid contents present on the CM. The proposed method of HS–AD also showed a significant reduction (percentage removal) of TS (66%), VS (61%), TCOD (78%), SCOD (92%) and TVFA (60%) as shown in Figure 2d.

Although the concept of HS–AD proposed here reduces the organic loading significantly, still the digestate contained a significant amount of organic matter. For example, the VS, SCOD and TVFA content of the digestate was still as high as 190 g/L, 21.91 g/L and 10.15 g/L, respectively. Additionally, useful nutrients were present within the liquid digestate. Henceforth, a post-treatment such as microalgae cultivation was proposed to have an additional treatment, to find the best way to utilize the nutrients present in the liquid digestate and convert them to an exportable quality since microalgae has several benefits or downstream applications, such as fertilizer, bio-products and so on (refer to Section 3.3). Therefore, microalgae post-treatment was chosen and discussed further.

### 3.2. Growth of Microalgal Strain Chlorella vulgaris CPCC 90

#### 3.2.1. Growth Curve of the Algal Strain

**Figure 3:**
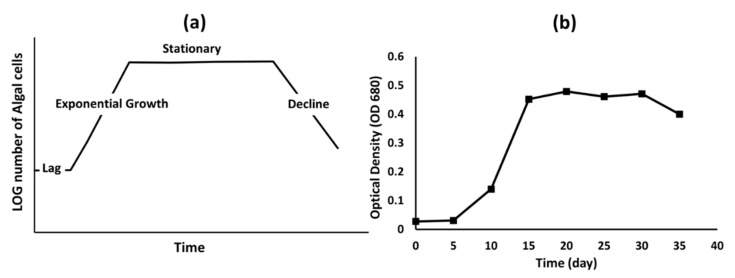
Algal growth curve. (**a**) Standard algal growth curve, (**b**) *Chlorella vulgaris* at 10% dilution of CM digestate.

Results showed that *Chlorella vulgaris* CPCC 90 was able to grow and utilize nutrients from 10% diluted anaerobically digested CM. Any higher concentration of CM leachate showed an inhibitory effect on algal growth as reported previously [34]. The growth of *Chlorella vulgaris* CPCC 90 was estimated by using two methods: hemocytometer and plate reader. However, the hemocytometer assay was not effective as a result of the presence of a lot of impurities in the CM, which interfered with the algal count; therefore, the plate reader spectrophotometer assay was used. The spectrophotometer plate reader was adjusted at 680 nm absorbance and the algal growth was monitored every five days over a period of 35 days as the incubation period. The growth curve of the algal strain on the CM is illustrated in Figure 3.

#### 3.2.2. Algal Incubation Experiments: Digestate Characteristics

Characteristics of the digestate before and after the algal incubation experiment are presented in Table 3. According to the basic formulation of microalgae (C_106_H_263_O_110_N_16_P) [8], it contains between 8.3% and 9.8% nitrogen and between 0.52% and 0.69% phosphorus in the biomass, which necessitate the availability of N and P for microalgae growth. Although most of the nitrogen in the CM is in organic form, under anaerobic conditions, this organic nitrogen could be converted to ammonia, which proved to be a more effective source of nitrogen for algal cell growth [35].

As seen in Table 3, considering the CM digestate removal efficiency, the microalgal strain presents great potential for consuming the nutrients and, thus, removing them from the digestate. The ammonia and total Kjeldal nitrogen removal rates were observed to be in the range of 99% and 85%, respectively. The total removal of nitrogen was higher mainly due to the pH fluctuations during the microalgae incubation period, which leads to ammonia gasification.

Carbon is another essential source for algal growth. COD removal rate was in the range of 44.6%, which confirms the capability of microalgal strain to consume organic substrates in the leachate as a carbon source and thus, their growth can effectively remove COD from the leachate sample.

In addition, the *Chlorella vulgaris* strain shows the ability to utilize volatile fatty acids (VFAs) as a carbon source for lipid accumulation and promote algal growth [36]. As presented in Table 3, the VFA removal rate was 74.2%, which is in accordance with previous studies [37].

### 3.3. Integration of AD and Microalgae Cultivation: Economic Considerations and Future Directions

The high nutrient content of CM suggests that these materials could be utilized as a source for producing further value-added products, providing more benefits for farming to be able to complete a sustainable circular bioeconomy. At the same time, the increasing interest in the commercial microalgae production industry could enhance the competition of this sector with the agricultural industry over using inorganic fertilizers as nutrients for growth. This will result in the fact that using inorganic fertilizers for producing microalgae may become an economically unviable option. Thus, using abundant nutrients that exist in animal manure to grow microalgae can provide a cheaper source of necessary nutrients, while helping the agricultural sector in managing surplus wastes. In this regard, utilizing CM as a nutrient source for growing microalgae has high potential, as it is rich in ammonia, phosphate, potassium, and other trace elements [38].This potential resulted in numerous studies integrating microalgae production with anaerobic digestion of animal manure. However a limited number of studies on the coupling of anaerobic digestion of CM with microalgae production have been reported. Duangjan et al. [26] investigated the production of a microalgal strain (*Scenedesmus* sp.) using different concentrations of anaerobically digested CM. Results showed that the optimum concentration for algal growth was 12–25%. The produced microalgae showed to be suitable for lipid production. Singh et al. [25] studied the production of microalgae consortia as a successful process to be integrated with anaerobic digestion of poultry litter. Their results confirmed that 6% (*v*/*v*) concentration of anaerobically digested poultry litter could be used as a microalgae growth medium. The algal biomass, being rich in protein and low in lipid, could make it a suitable candidate for use as an animal feed supplement [28], provided that the pathogens and other contaminations are eliminated.

Given the high nutrient content of CM, proper management of this valuable “resource” financially contributes to the sustainable agricultural practices by providing valuable commodities for farmers. Theoretically, harvesting the available nutrients in CM by exploiting the existing technologies is sufficient to produce enough by-products for the farmers and generate more income. As reported by Hansen [39], a typical poultry farm usually produces between 19 and 46 kg of manure annually per chicken with an average of 33 kg/y. The average nutrient content per ton of CM is presented in Table 4 [39].

A simple calculation shows that the amount of nitrogen, phosphorus and potassium disposed annually in the form of CM is in the range of 0.53–0.89 kg, 0.63–0.94 kg, and 0.42–0.6 kg per animal, respectively, which could add up to around 7 tons of nitrogen and 8 tons of phosphorus annually for an average-size farm (10,000 birds). This high volume of nutrients is often considered a hitch, as it has to be either disposed of or stored at a proper place for later use. However, in this work, we suggest considering manure as a by-product of the agricultural industry. Coupling the AD process and microalgae cultivation could result in a framework that generates high-value by-products to be offered on the market.

**Figure 4:**
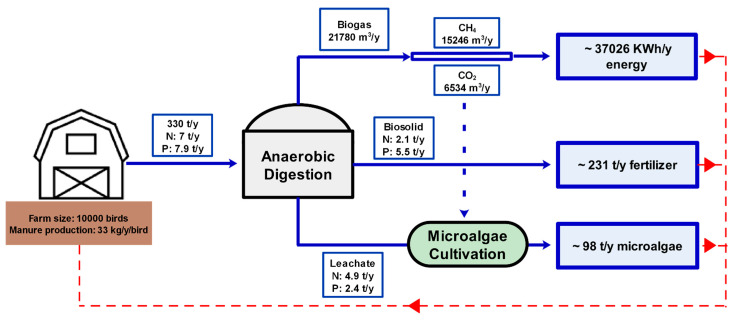
Nutrient mass balance in a closed-loop recycling framework in a poultry farm.

Figure 4 shows the theoretical value of the valuable bio-products that can be generated following the proposed AD–microalgae coupling framework in this work.

According to the proposed framework, on average, 330 tons of manure can be produced annually from a poultry farm that consists of 10,000 birds. This manure can be treated by the AD process and produce approximately 21,780 m^3^ biogas annually. Biogas can be used to produce 37,026 KWh/y energy in the form of electricity and heat that can be used internally by the farm itself, and the excess amount can be sold in the market. On the other hand, in the given scenario, AD can produce about 230 t of biosolids annually, which can be used as a fertilizer or a soil enhancer. The leachate of the AD process, conventionally, is treated with further treatment technologies and discharged. However, this framework suggests that the amount of nutrient available in this leachate is around 4.9 t/y for N and 2.4 t/y for P, which is enough to produce around 100 t/y microalgae [12], which has been proved to have high potential for various downstream applications.

In spite of the technical feasibility of producing microalgae with CM waste, the energy and economic feasibility aspects need more improvement to be considered as a viable option, especially when considering microalgae for the purpose of biodiesel production [40,41]. However, considering other scenarios for utilizing microalgae in the agriculture sector, such as extracting and utilizing of concentrated proteins as animal feed, will make this integration a profitable and sustainable solution [42]. Favorably, the feasibility of using microalgae as animal feed has been extensively studied and, in some cases, it has been commercialized. This scenario would bring double benefits since the supplementation of microalgae to the animal diet can also have a positive effect on human health, as those animals are used as food for humans. Previous research shows that when pigs were fed algal species with rich iodine content, this led to the production of pork rich in iodine to be consumed by humans [43]. Current research [44] shows that when the pigs diet was supplemented by microalgae rich in docosahexaenoic acid (DHA), this led to the production of DHA-enriched pork, which may have a role in overcoming the omega-3 fatty acids nutrition gap and subsequently enhancing public health. When the microalgae *Aurantiochytrium limacinum* was used as a food supplement for lactating dairy cows (105 g/head/d), this resulted in production of milk rich in DHA without any negative effects on the cows [45]. It was also found that when the microalgae Spirulina replaced 50% of the soy protein in poultry diets, there was an obvious improvement in the quality of the produced meat; however this replacement led to changes in the color of the meat [46].

**Figure 5:**
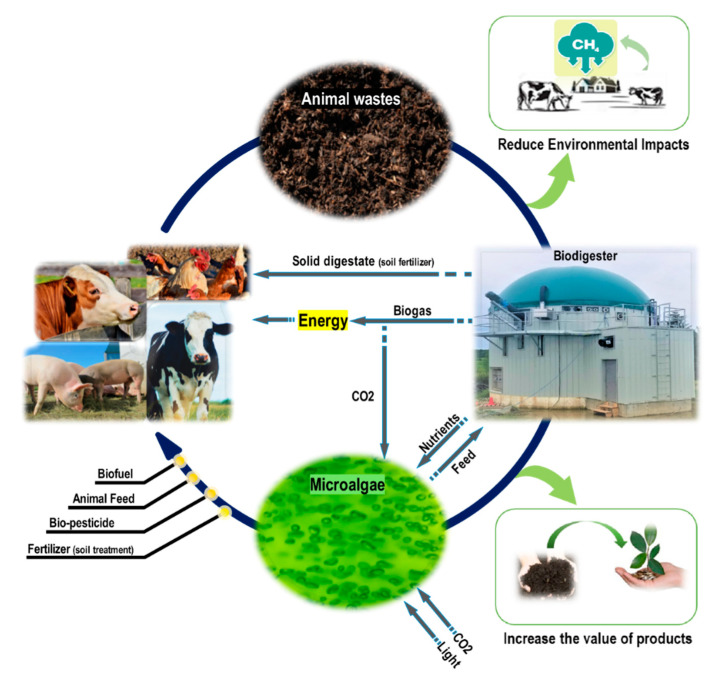
Agricultural waste management toward circular bioeconomy.

Despite these favorable results, further research is necessary to optimize the dilution factor and to test the effect of using microalgae/microalgae-bacteria consortia for better adaptation to high-N content waste. Figure 5 is a schematic demonstration of the closed-loop nutrient recovery system in managing agricultural organic wastes toward a circular bioeconomy concept.

This schematic represents the benefits of the proposed coupling solution. Animal waste is managed through AD treatment, which can reduce the environmental impact of the industry by converting harmful products to two beneficial forms, biogas and biosolids. Conventionally, biogas and biosolids have been recycled back to the farm in the form of energy and soil fertilizer; however, more valuable nutrients in the leachate is being neglected. In this suggested framework, by coupling the AD with microalgae, these nutrients are being kept in the loop and will generate high value by-products in the form of microalgae. Ultimately, microalgae can potentially be used in various forms, as described in Figure 5, by the farmers, which has an attractive commercial value. Moreover, providing the required CO_2_ and energy for the microalgae cultivation step by the AD process will make this nutrient recovery closed-loop framework, a robust and self-sufficient practice.

## 4. Conclusions

Among different treatment technologies for CM waste management, the AD process is a promising technique. The proposed closed-loop recirculation and percolation mode of treating CM showed a very robust and efficient method for the treatment of CM and it can operate not only without any digester failure but also produces very high quality methane despite having high ammonia and solid contents in the raw CM. Preliminary results of microalgae cultivation indicate that *Chlorella vulgaris* CPCC 90 was able to grow and utilize nutrients from 10% sterile diluted CM at low temperature 20 ± 1 °C. However, high-N content has an inhibitory effect on growth of microalgae for samples with a dilution factor higher than 30%.

## Figures and Tables

**Table 1:** Physicochemical characteristics of raw chicken manure (CM) and liquid digestate (leachate).

Parameter	Raw CM	Liquid Digestate (Leachate)
Alkalinity (mg/L)	5.393	21,405
pH	7.73	7.76
NH3-N (mg/L)	5913	5314
TKN (mg/L)	25,652	6197
Total solids (*w*/*w* %)	69.87	2.8
Volatile solids (*w*/*w* %)	61.09	1.44
Total COD (mg/L)	864,375	35,557
Soluble COD (mg/L)	291,149	30,685
Volatile fatty acids (mg/L)	25,456	11,812

**Table 2:** Average values of a two-stage digester.

Cycle Length	Cumulative Biogas (L)	Cumulative Methane (L)	Methane Content (%)	SMY (L CH_4_/ g VS)	OLR (gVS/L.d)
70 days	578 ± 42	382 ± 31	70 ± 11	0.46 ± 0.05	8.7

**Table 3:** Digestate characteristics from the algal incubation experiments.

Parameter	Initial CM Digestate Concentration *	Final Value of CM Digestate after Microalgal Treatment	Removal Efficiency (%)
NH3-N (mg/L)	531	3	99.4
TKN (mg/L)	619	91	85.3
Total COD	3555	1970	44.6
Volatile fatty acids	1181	304.6	74.2
Total solid (*w*/*w* %)	0.28	0.158	43.5
Volatile solid (*w*/*w* %)	0.144	0.112	22.2

* Note: ‘Substrate initial value’ represents the concentration of each item in 10% diluted CM liquid digestate (leachate); and the ‘Final value’ represents the concentration of the resultant digestate after the 35 d incubation period, which is the difference between ‘Algae+CM leachate’ and ‘Algae alone’.

**Table 4:** Average nutrient content per ton of CM.

Manure Type	Nitrogen (kg/t)	Phosphorus (kg/t)	Potassium (kg/t)
Broiler litter	26.7	28.5	18
Hens (laying)	16	19	12.7
Average	21.3	24	15.4
